# The agreement between ultrasound-determined joint inflammation and clinical signs in patients with rheumatoid arthritis

**DOI:** 10.1186/s13075-019-1892-0

**Published:** 2019-04-17

**Authors:** Xiaoying Sun, Xuerong Deng, Wenhui Xie, Liujun Wang, Yu Wang, Zhuoli Zhang

**Affiliations:** Department of Rheumatology and Clinical Immunology, Peking University First Hospital, No. 8 Xishiku Street, West District, Beijing, 100034 China

**Keywords:** Rheumatoid arthritis, Ultrasound, Synovitis, Tenosynovitis, Peritendinitis, Tenderness, Swelling

## Abstract

**Background:**

Ultrasound (US) is sensitive for detecting joint and tendon inflammation in patients with rheumatoid arthritis (RA). So far, which grade of abnormalities on US corresponds to clinical manifestations is unclear. This study aimed to investigate the agreement between US-detected joint inflammation and clinical signs (joint swelling and tenderness).

**Methods:**

In this cross-sectional study, 22 joints of the wrists and hands were, respectively, evaluated by physical examination (PE) and ultrasound in RA patients. Gray scale (GS) and power Doppler (PD) of synovitis, detected by ultrasound, were graded by semi-quantitative scoring systems (0–3). Tenosynovitis and peritendinitis were assessed qualitatively (0/1).

**Results:**

A total of 258 consecutive RA patients were included, with median disease duration of 57 months and mean Disease Activity Score based on 28 joints (DAS28)-ESR/DAS28-CRP of 4.47/3.99. In a total of 5676 joints assessed, the overall concordance rate between positive clinical signs and ultrasound-determined joint inflammation was fair (*κ* = 0.365, *p* < 0.01). In wrists, joint tenderness showed higher *κ* coefficient (*κ* = 0.329, *p* < 0.01) with ultrasound-determined joint inflammation than swelling (*κ* = 0.263, *p* < 0.01); however, swelling showed higher κ coefficient (*κ* = 0.156–0.536, *p* < 0.01) with ultrasound-determined joint inflammation than tenderness (*κ* = 0.061–0.355, *p* < 0.01) in metacarpophalangeal (MCP) and proximal interphalangeal (PIP) joints. Synovitis had consistently higher agreement with tenderness and swelling than tenosynovitis/peritendinitis. Tenderness and swelling had the highest *κ* coefficient with GS ≥ 1 synovial hyperplasia in most MCP and PIP joints, while with GS ≥ 2 synovial hyperplasia in wrists. For all 22 joints, PD ≥ 1 synovitis had the highest κ coefficient with clinical tenderness and swelling.

**Conclusions:**

Synovitis had better agreement with clinical signs than tenosynovitis/peritendinitis. Joint swelling showed better agreement with US-determined inflammation than tenderness for MCP and PIP joints, while the opposite for wrists. Both tenderness and swelling are more likely to correspond to GS ≥ 2 for wrists, GS ≥ 1 for MCP and PIP joints, and PD ≥ 1 for any joint.

## Background

Rheumatoid arthritis (RA) is an inflammatory disease characterized by chronic intra-articular and peri-articular synovial inflammation associated with joint destruction and function impairment [1]. Patients with RA have polyarthritis appearing as joint swelling and tenderness. These signs are identified as joint inflammation by clinicians through physical examination (PE). Swollen and tender joint counts are essential parameters to access clinical disease activity and further formulate the treatment target in RA patients, including the Disease Activity Score based on 28 joints (DAS28) [2], Clinical Disease Activity Index (CDAI), Simplified Disease Activity Index (SDAI) [3], American College of Rheumatology (ACR) response criteria [4], and the new Boolean-based remission criteria [5], in both clinical practice and trials. But joint tenderness is to some extent subjective depending on the judgment of an individual patient and clinician. Besides, it is sometimes difficult to accurately determine joint swelling by PE alone if the patient accompanied by various factors such as obesity and edema. Ultrasound (US) is a non-invasive, inexpensive, and free-of-radiation imaging technique allowing a quick and sensitive assessment of soft tissue inflammation. US shows superior sensitivity and inter-observer reliability in reflecting joint inflammation in RA patients compared to PE [6–8], and equivalent accuracy in detecting pathological abnormalities as magnetic resonance imaging (MRI) at finger level [9]. US examination can be used to visualize anatomically involved joints with synovial hypertrophy/effusion using the gray scale (GS) mode, to assess the degree of synovial inflammation, and predict subsequent joint damage using the power Doppler (PD) mode [10]. At present, the most frequently employed semi-quantitative scoring system to grade the synovial hypertrophy/synovitis is proposed by Szkudlarek et al. [11]. Both GS and PD are graded on a scale of 0–3 according to the severity of synovial hypertrophy and vascularization. Several single-center studies and meta-analyses have demonstrated a predictive value of PD positivity for flare and progressive bone erosion in patients with RA [12–15]. But it remains unclear which grade of GS indicates a pathological finding [16–20].

Wrists, metacarpophalangeal (MCP), and interphalangeal (PIP) joints are the most frequently affected in RA [21]. Pathologic findings in these joints are considered to be representative and precursors of overall joint damage. The aim of this study was to investigate the agreement between clinical-detected signs and US features of joint inflammation in wrists and hands and further determine the grades of GS synovial hyperplasia and PD synovitis which correspond to the presence of tenderness and swelling in an individual joint in RA patients.

## Methods

### Patients

This is a cross-sectional study which consecutively enrolled 258 patients with RA who visited the rheumatology clinic of Peking University First Hospital between February 2014 and May 2017. All these patients fulfilled the 2010 ACR/European League Against Rheumatism (EULAR) classification criteria and were more than 18 years old [22]. All patients had at least 1 tender or swollen joint out of 22 joints (bilateral wrists, MCP1–5, and PIP1–5 joints). Both clinical and US data of all patients were collected and analyzed. The usage of conventional synthetic disease-modified anti-rheumatic drugs (csDMARDs) (methotrexate, leflunomide, hydroxychloroquine, sulfasalazine); glucocorticoids; biological (b) DMARDs (adalimumab, etanercept, abatacept, rituximab); and non-steroidal anti-inflammatory drugs (NSAIDs) were recorded. Those patients with other comorbidities, for instance, psoriatic arthritis, gout, history of trauma, and/or joint replacement of a wrist/finger, with obvious joint deformity or mutilation, were excluded in this study. This study was conducted in accordance with the Declaration of Helsinki and was approved by Institutional Medical Ethics Review Boards of Peking University First Hospital. The informed consent was obtained from each patient on entry.

### Joint and laboratory assessment

Independent joint assessment for tenderness and swelling was performed by a rheumatologist who was blinded to both clinical and ultrasound data. Tender and swollen joints among 28 areas (bilateral shoulders, elbows, wrists, knees, MCPs, and PIPs) were counted. The patient’s global assessment (PGA; 0–100 mm visual analog scale) and evaluator’s global assessment (EGA; 0–100 mm visual analog scale) were rated individually by each patient. Serum concentrations of C-reactive protein (CRP) and erythrocyte sedimentation rate (ESR) were measured. Disease activity was assessed by DAS28-ESR, DAS28-CRP, CDAI, and SDAI.

### US assessment

Ultrasound machine LOGIQ E9 and ML 6–15-Hz linear probe were used to scan 22 joints (bilateral wrists, MCP1–5, and PIP1–5 joints) for all patients, by both GS and PD modes. The US examinations were performed in a standardized manner according to the EULAR guidelines for musculoskeletal US in rheumatology [23]. The PD settings included a pulse repetition frequency (PRF) of 500–750 Hz, low wall filter, and Doppler gain, which were adjusted to produce the higher sensitivity, but avoiding random noise visualization. The interpretation of lesions was based on the published literature of Outcome Measures in Rheumatology Clinical Trials (OMERACT) [24]. US-determined joint inflammation was defined as synovitis and/or tenosynovitis/peritendinitis. Synovitis was assessed by semi-quantitative scoring systems (0–3) proposed by Szkudlarek et al. [11], tenosynovitis (wrists and flexor tendons in fingers)/peritendinitis (extensor tendons in fingers) was qualitatively scored by 0/1. For synovitis, the maximum GS and PD grade recorded on volar and dorsal aspects for a given joint region was recorded as the GS and PD grade for the respective joint. Synovitis was defined as GS ≥ 1 and/or PD ≥ 1. Tenosynovitis was evaluated in six extensor compartments and flexor tendons within the wrist region. The presence of at least one extensor compartment or any flexor tendon in the wrist was considered to be positive. Peritendinitis was defined as the inflammation surrounding extensor tendons in fingers lacking of tendon sheaths. Both tenosynovitis and peritendinitis were defined as the presence of GS or PD signal. Either tenosynovitis or peritendinitis at the level of the MCPs and PIPs was regarded as positive finding. All the US scanning was done by one of three ultrasonographers with over 5 years of experience in maneuvering musculoskeletal ultrasound. In all patients, the clinical examination and US were blindly assessed on the same day. Five patients were randomly selected to test the inter-observer reliability of the US evaluation between the operators and analyzed by intra-class correlation coefficient (ICC). The inter-observer reliability for GS was 0.986 (95% CI 0.981–0.990) and 0.988 (95% CI 0.983–0.991) for PD, indicating the reliability was excellent.

### Statistical analysis

Statistical analysis was performed with SPSS 21.0. For the descriptive analyses, continuous variables were presented as mean and standard deviation (SD) if normally distributed and median and interquartile range (IQR) if non-normally distributed. Independent *t* test and Wilcoxon signed test were applied, as appropriate. Dichotomous variables are presented as frequencies and percentages and were compared by *χ*^2^ test. Absolute agreements and Cohen’s kappa (*κ*) between clinical and sonographic findings were calculated. The *κ* coefficients were divided as follows: 0–0.20 = poor, 0.20–0.40 = fair, 0.40–0.60 = moderate, 0.60–0.80 = good, and 0.80–1.00 = excellent. Kappa values represent a measure of by how much the observed agreement exceeds agreement by chance. Kappa values tend to be low if data is skewed, even if agreement is very good. *P* values < 0.05 were considered statistically significant.

## Results

### Demographics and clinical characteristics of patients

The characteristics of the 258 enrolled patients are illustrated in Table 1. Their median age was 51.2 years and median disease duration was 57 months, with 83.33% being females. The mean (SD) DAS28-ESR and DAS28-CRP were 4.47 ± 1.62 and 3.99 ± 1.51, respectively. The median (IQR) CDAI and SDAI were 14 (8, 24) and 15.38 (8.30, 26.83), respectively. The median swollen joint count (SJC)-22 and tender joint count (TJC)-22 were 2 (0, 5) and 3 (1, 6), respectively. The majority of patients were treated with csDMARDs, including methotrexate (62.02%), hydroxychloroquine (37.21%), leflunomide (27.13%), and sulfasalazine (4.65%). Biologic DMARDs were used in 12 patients (4.65%). Mono, 2 and ≥ 3 DMARDs combination therapy were observed in 32.56%, 36.05%, and 11.24% of patients. Fifty-eight (22.48%) patients were receiving concurrent steroid therapy, including oral glucocorticoid of 41 (15.89%) patients and intramuscular injection of glucocorticoid within 14 days of 17 patients (6.59%). Twenty-three (8.91%) patients were treated with non-steroidal anti-inflammatory drugs (NSAIDs) on demand. The median daily dose of glucocorticoid was 10 mg (prednisone equivalent).

**Table 1:** Demographics and clinical characteristics of 258 RA patients

Parameters	Values
Age (years), mean ± SD	51.23 ± 13.74
Female, *n* (%)	215 (83.33)
Disease duration(months), median (IQR)	57 (14.75, 121.75)
Positive RF, *n* (%)	165 (73.01)
Positive ACPA, *n* (%)	153 (87.43)
TJC-22, median (IQR)	3 (1.6)
SJC-22, median (IQR)	2 (0.5)
ESR (mm/h), median (IQR)	29 (16.49)
CRP (mg/L), median (IQR)	9.24 (3.57, 27.60)
Patient’s global assessment (mm), median (IQR)	50 (30, 70)
Evaluator’s global assessment (mm), median (IQR)	40 (20, 60)
DAS28-ESR, mean ± SD	4.47 ± 1.62
DAS28-CRP, mean ± SD	3.99 ± 1.51
CDAI, median (IQR)	14 (8, 24)
SDAI, median (IQR)	15.38 (8.30, 26.83)
HAQ DI, median (IQR)	0.40 (0.10, 0.90)
Individual of csDMARD use	
Methotrexate, *n* (%)	160 (62.02)
Hydroxychloroquine, *n* (%)	96 (37.21)
Leflunomide, *n* (%)	70 (27.13)
Sulfasalazine, *n* (%)	12 (4.65)
bDMARD use (%)	12 (4.65)
Number of DMARDs	
0, *n* (%)	52 (20.16)
1, *n* (%)	84 (32.56)
2, *n* (%)	93 (36.05)
≥ 3, *n* (%)	29 (11.24)
Glucocorticoid use (%)	
Oral glucocorticoid use, *n* (%)	41 (15.89)
Oral glucocorticoid dose (mg/day), median(IQR)	10 (5.10) (prednisone equivalent)
Recent intramuscular injection of glucocorticoid (within 14 days), *n* (%)	17 (6.59)
NSAIDs on demand, *n* (%)	23 (8.91)

*Abbreviations: RF* rheumatoid factor, *ACPA* anti-citrullinated peptide antibody, *TJC-22* tender joint count in bilateral wrists, MCPs and PIPs joints, *SJC-22* swollen joint count in bilateral wrists, MCPs and PIPs joints, *ESR* erythrocyte sedimentation rate, *CRP* C-reactive protein, *DAS28-ESR* Disease Activity Score based on 28 joint count and ESR, *DAS28-CRP* Disease Activity Score based on 28 joint count and CRP, *CDAI* Clinical Disease Activity Index, *SDAI* Simplified Disease Activity Index, *HAQ DI* Health Assessment Questionnaire disability index, *csDMARDs* conventional synthetic disease-modified anti-rheumatic drugs, *bDMARD* biological disease-modified anti-rheumatic drugs, *NSAIDs* non-steroidal anti-inflammatory drugs

### Frequencies of tender or swollen joints by PE and inflammation detected by US

In a total of 5676 joints assessed, there were 968 swollen joints (17.05%) and 1296 tender joints (22.83%), while on ultrasonography GS synovial hyperplasia was present in 801 (14.11%) joints and PD synovitis in 476 (8.39%) joints (Table 2). There were 217 (84.11%) and 191 (74.03%) patients who presented tenderness and swelling of at least one joint, respectively. GS synovial hyperplasia and PD synovitis were presented in 220 (85.27%) and 160 (62.01%) patients, while GS and PD tenosynovitis/peritendinitis were detected in 67 (25.97%) and 43 (16.67%) patients, respectively. Joint inflammation detected by both PE and US most frequently occurred to the wrists. The tender or swollen joints detected by PE was more frequent in PIP joints than MCP joints, whereas the US-determined joint inflammation was more present in MCP joints than PIP joints. In 22 joints, the highest prevalence of tenosynovitis/peritendinitis was observed in the wrist. In wrists, tenosynovitis was observed more commonly in extensor compartments instead of flexor tendons. Specifically, the top three extensor compartments were the sixth compartment (extensor carpi ulnaris) (9.50%), the fourth compartment (extensor digitorum and extensor indicis) (3.49%), and the second compartment (extensor carpi radialis longus and brevis) (3.29%).

**Table 2:** Frequencies of PE detected tenderness or swelling and US-detected inflammation in wrist and finger joints

Joints	PE detected tenderness or swelling (%)		US-detected inflammation (%)		
	Tenderness	Swelling	GS synovial hyperplasia	PD synovitis	Tenosynovitis/peritendinitis
MCP1	19.38	11.67	12.92	6.25	0.00
MCP2	24.79	22.29	20.21	13.96	0.00
MCP3	19.58	17.71	15.83	10.63	1.16
MCP4	13.13	11.04	10.00	6.88	0.58
MCP5	15.42	12.71	13.54	6.88	0.19
PIP1	20.63	13.75	3.54	1.46	0.39
PIP2	27.50	20.63	3.13	1.04	1.16
PIP3	32.50	22.29	7.50	3.13	2.32
PIP4	25.00	16.67	5.83	2.92	1.35
PIP5	22.92	16.88	3.96	1.46	0.58
Wrist	49.17	36.04	66.46	43.75	13.37
At joint level	22.83	17.05	14.11	8.39	NA
At patient level	84.11	74.03	85.27	62.01	25.97

*Abbreviations: GS* gray scale, *PD* power Doppler, *MCP* metacarpophalangeal, *PIP* proximal interphalangeal, *PE* physical examination, *US* ultrasound, *NA* not applicable

### Various grades of GS synovial hyperplasia and PD synovitis, as a proportion of all findings

**Fig. 1:**
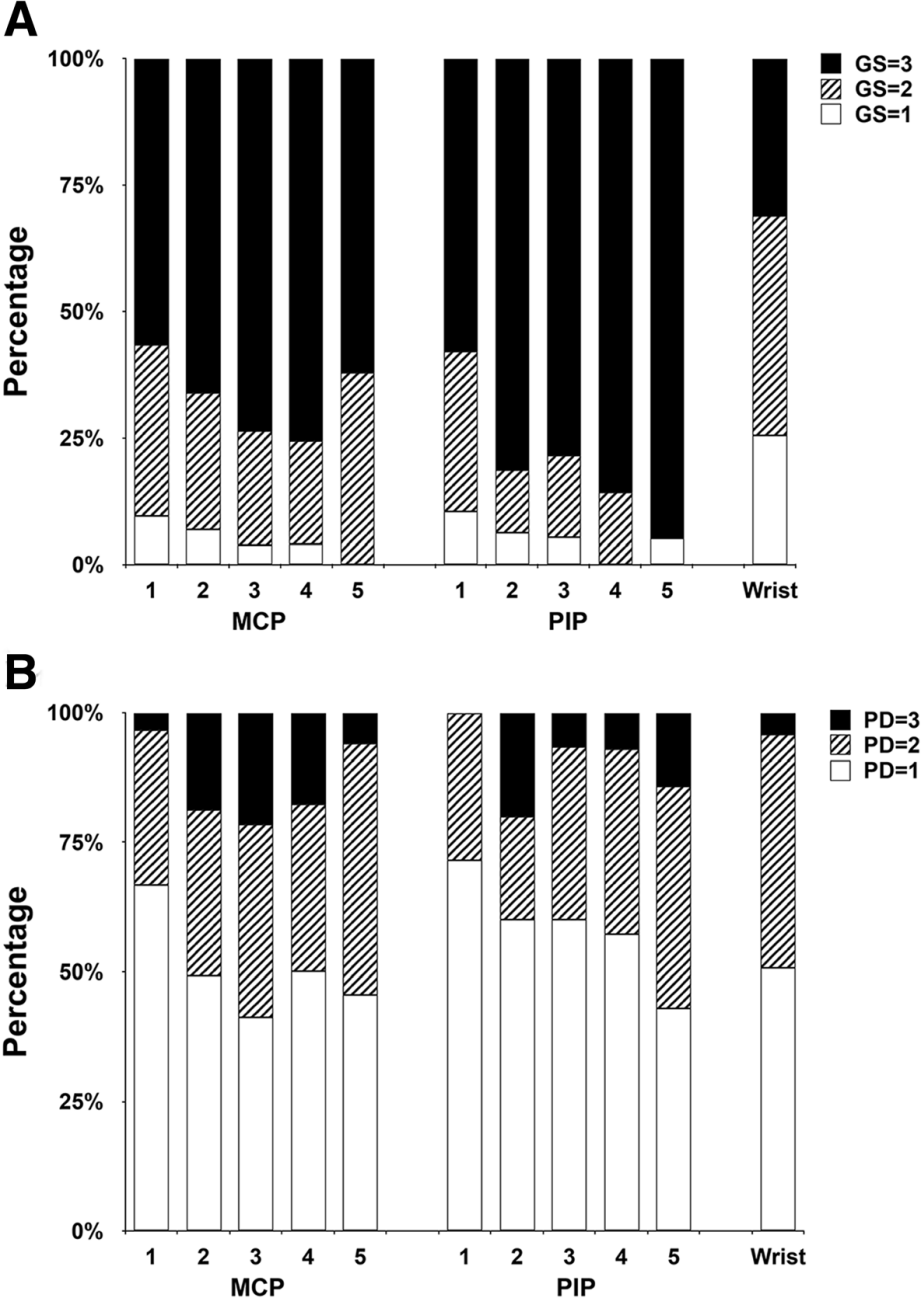
Various grades of GS synovial hyperplasia and PD synovitis, as a proportion of all findings. **a** Grade 1, grade 2, and grade 3 GS synovial hyperplasia, as a proportion of all findings for MCP1–5, PIP1–5 and wrist joints. **b** Grade 1, grade 2, and grade 3 PD synovitis, as a proportion of all findings for MCP1–5, PIP1–5, and wrist joints. *Abbreviations*: *GS* gray scale, *PD* power Doppler, *MCP* metacarpophalangeal, *PIP* proximal interphalangeal

Interestingly, the majority (432 [53.93%]) of the 801 GS-positive joints exhibited grade 3 of synovial hyperplasia, while GS grade 2 was presented in 261 joints (32.58%) and GS grade 1 in only 108 joints (13.48%). GS grade 3 finding was more frequently observed in MCP and PIP joints than in wrists. In contrast to these GS findings, the severity of PD synovitis was quite different. Among the 476 joints with PD synovitis, PD grade 1 was presented in 242 (50.84%) joints, grade 2 in 188 (39.50%) joints, and grade 3 in only 46 (9.66%) joints. The frequencies of grades 1, 2, and 3 on GS and PD were comparable among the three joint regions examined and between individual MCP and PIP joints of all different digits (Fig. 1a, b).

### The concordance between tenderness or swelling by PE and US-determined inflammation

The agreement of clinical signs and US-determined inflammation in all joints was fair (78.38%, *κ* = 0.365, *p* < 0.01) (Table 3). The highest κ coefficient between clinical signs and ultrasound-determined inflammation was seen in MCP3 joint (82.36%, κ = 0.458, p < 0.01), followed by MCP2 joint (78.49%, κ = 0.426, *p* < 0.01). Compared with PIP joints, MCP joints showed higher agreements and concordance rates. In PIPs, there were more tender/swollen joints without US-determined inflammation (PE+/US−) than those non-tender/swollen joints but with US-detected inflammation (PE−/US+). The differences between clinical signs and US-determined inflammation in both wrists and the MCP joints were insignificant.

**Table 3:** The agreement between clinical findings and ultrasound detected inflammation, for wrist and finger joints

Joint	Number of joints				Agreement (%)	Kappa
	PE+/US+	PE+/US−	PE−/US+	PE−/US−		
MCP1	37	69	25	385	81.78%	0.340^*^
MCP2	71	81	30	334	78.49%	0.426^*^
MCP3	58	70	21	367	82.36%	0.458^*^
MCP4	30	50	22	414	86.05%	0.379^*^
MCP5	39	54	28	395	84.11%	0.396^*^
PIP1	17	96	4	399	80.62%	0.199^*^
PIP2	15	137	7	357	72.09%	0.106^*^
PIP3	38	138	10	330	71.32%	0.226^*^
PIP4	26	108	9	373	77.33%	0.224^*^
PIP5	16	114	6	380	76.74%	0.148^*^
Wrist	237	41	107	131	71.32%	0.411^*^
Total	584	958	269	3865	78.38%	0.365^*^

*Abbreviations: MCP* metacarpophalangeal, *PIP* proximal interphalangeal, *PE+* presence of tenderness or swelling by physical examination, *US+* GS ≥ 1 or PD ≥ 1 for synovitis or tenosynovitis/peritendinitis

*p < 0.01

**Fig. 2:**
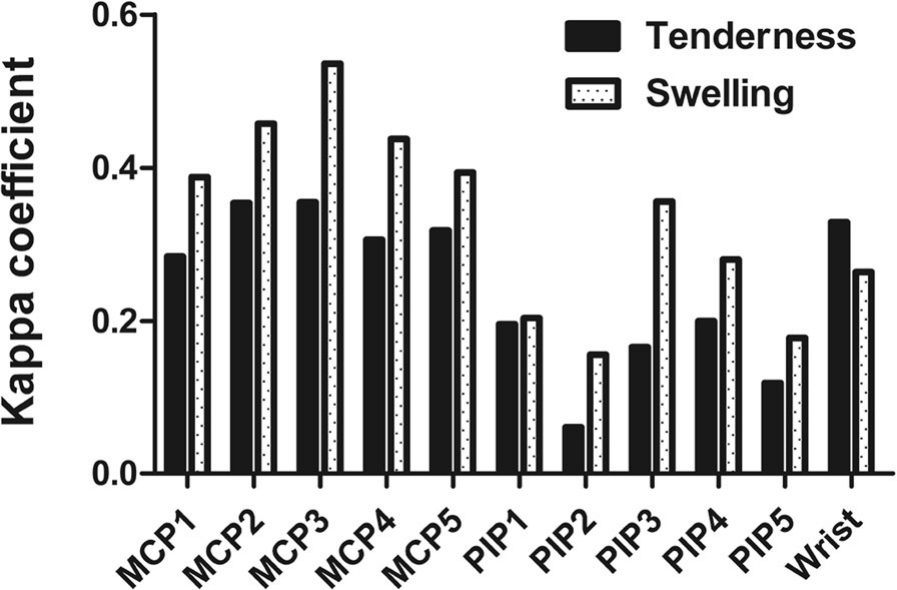
Concordance (κ coefficient) between joint tenderness or swelling by PE and US-determined inflammation for each joint. *Abbreviations*: *MCP* metacarpophalangeal, *PIP* proximal interphalangeal

In wrists, joint tenderness showed higher *κ* coefficient (*κ* = 0.329, *p* < 0.01) with ultrasound-determined inflammation than swelling (*κ* = 0.263, *p* < 0.01), while on the contrary, swelling showed higher *κ* coefficient (*κ* = 0.156–0.536, *p* < 0.01) with ultrasound-determined inflammation than tenderness (*κ* = 0.061–0.355, *p* < 0.05) in MCP and PIP joints (Fig. 2).

Tenosynovitis/peritendinitis (*κ* = 0.070/0.092, *p* < 0.01) detected by ultrasound showed much lower agreement with tenderness/swelling than synovitis (*κ* = 0.292/0.363, *p* < 0.01). The highest *κ* coefficients between clinical tenderness and swelling and tenosynovitis/peritendinitis were seen in wrists (*κ* = 0.119/0.172, *p* < 0.01). In part MCPs and PIPs, tenosynovitis/peritendinitis did not significantly correspond with tenderness/swelling (*p* > 0.05).

### Concordance (*κ* coefficient) between clinical tender or swollen joint and GS or PD grades for each joint region

**Fig. 3:**
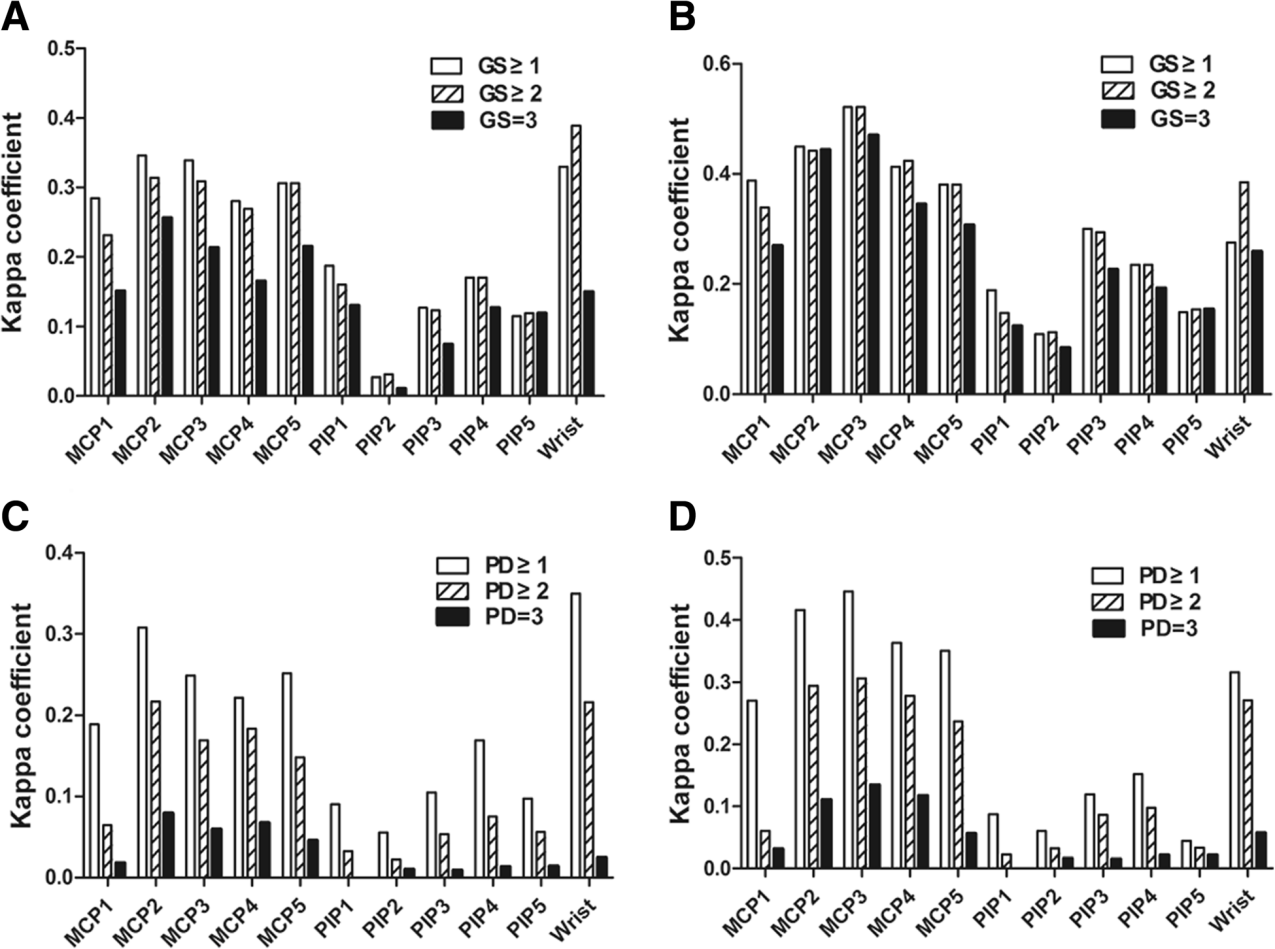
Concordance (κ coefficient) between tender or swollen joint and GS/PD grades for each joint region. **a** *κ* coefficient between tenderness and GS grade. **b** *κ* coefficient between swelling and GS grade. **c** *κ* coefficient between tenderness and PD grade. **d** *κ* coefficient between swelling and PD grade. *Abbreviations*: *GS* gray scale, *PD* power Doppler, *MCP* metacarpophalangeal, *PIP* proximal interphalangeal

Both GS and PD grades corresponded to clinical tenderness and swelling with different coefficients in different joints (Fig. 3). Joint tenderness and swelling had the highest *κ* coefficient with GS ≥ 1 synovial hyperplasia in most MCP and PIP joints, while with GS ≥ 2 synovial hyperplasia in wrists. For all 22 joints, PD ≥ 1 synovitis had the highest *κ* coefficient with both tenderness and swelling of joints by PE.

## Discussion

Eradication of joint inflammation is the prerequisite for retarding the progression of bone destruction in RA; therefore, accurate assessment of the disease activity has become critical. All clinical indicators for the evaluation of disease activity include TJC and SJC, which are obtained by PE. However, the perception of pain is highly subjective and may be influenced by a number of issues, including socio-cultural factors [25–27]. Moreover, Basu et al. firstly provided objective neuroimaging evidence that RA is a mixed pain state displaying characteristics of central sensitization [28]. Previous studies showed that as many as 50% of patients continue to report clinically significant levels of pain despite excellent control of their peripheral inflammation [29, 30]. Joint swelling may also be due to synovitis or non-vascular soft tissue, such as bony joint swelling in osteoarthritis, fat tissue, and subcutaneous edema. Additionally, high intra- and inter-observer variability in PE is inevitable [31–33]. Therefore, it should be highly concerned that joint inflammation in some RA patients may be inaccurately judged due to the limitations of PE. So far, the relative importance of clinical signs and whether it depends on joint region remains unclear. Ultrasound assessment of the joints shows superior sensitivity and inter-observer reliability in detecting joint inflammation compared to PE in RA population. However, which grade of abnormalities on US corresponds to clinical manifestations and is detrimental need to be clarified.

In the calculation of DAS28, the tender joint count weights twice of the swollen joint count [34]. Similarly, other clinical scores imply the same hierarchy of importance. However, in recent years, researchers have proposed the opposite ideas. Ceponis et al. showed the agreement between intra-articular PD signal and joint swelling was better than joint tenderness to palpation [35]. Similarly, Krabben et al. indicated the association of inflammation on MRI with swollen joints was stronger than with tender joints, illustrating the presence of swelling might be more significant than tenderness [36]. Besides, recent studies confirmed that compared to joint tenderness, swelling is the true predictor of subsequent radiographic progression in RA [37–39]. Some patients exhibit persistent chronic synovitis, manifesting as joint swelling and which may not be accompanied by pain. Thus, DAS28 has been challenged as a good assessment tool of disease activity [37]. In our study, joint swelling showed better agreement with ultrasound-determined inflammation than tenderness for MCP and PIP joints, which corresponded to the view proposed in recent studies where swelling contributes more to the joint inflammation than tenderness. On the contrary, tenderness showed better consistency with inflammatory lesions under ultrasound than swelling in wrists. To our knowledge, this has not been previously described. This discrepancy could be attributed to that the wrist, as a complex joint, is composed of multiple bones and joints, which intercommunicate through a common synovial cavity. The joint capsule is loose and thin on the dorsal side and can contain synovial folds. The relatively scattered distribution of synovial tissue may make the swelling of wrists uneasily judged well. Conversely, MCP and PIP joints are small and superficial with relatively closed joint capsule; therefore, swelling is easily found.

In wrists, synovial hyperplasia evaluated by GS and synovitis by PD on ultrasound were more sensitive than tenderness and swelling in reflecting joint inflammation. While for PIPs, the tenderness and swelling detected by PE were much more frequent than the ultrasound-determined inflammation. PIPs showed lower agreements and concordance rates between clinical signs and US-determined inflammation than MCPs and wrists. Some possible reasons may contribute to this result. Firstly, compared to wrist and MCP joints, PIP joints are much smaller, more superficial with closed joint capsule; therefore, clinical signs can be more easily detected in PIP joints. Secondly, PIPs are more involved in osteoarthritis, in which joint tenderness or swelling is mainly contributed by osteophytes rather than synovitis or tendon inflammation. Thirdly, the most severe part of the joint may be missed by US as it is a two-dimensional image.

Except for synovitis, a swollen or tender joint can also be due to coexisting or alone tenosynovitis/peritendinitis. Previous results indicated better agreement of synovitis with clinical signs than with tenosynovitis/peritendinitis [36]. Compared to MCPs and PIPs, the tenosynovitis/peritendinitis of the wrists not only had the highest positive ratio, but higher consistency with PE. The tenosynovitis/peritendinitis of the wrist which is more easily detected than relatively small MCP and PIP joints by ultrasound may be the reason.

Currently, a cut-off defining active disease from a GS point of view is not available and the optimum cut-off value to distinguish RA patients from healthy individuals’ synovial thickness varies in different joints [40]. Compared to MRI, the sensitivity and specificity of GS (cut-off ≥ 1) for detecting synovitis also vary greatly among the different joint locations [41]. In the present study, tenderness and swelling were best consistent with GS ≥ 2 synovial hyperplasia in the wrists. Ogishima et al. reported that the wrists were more prone to developing subclinical synovitis than PIP and MCP joints [13], indicating the inaccurate clinical evaluations and the need for US as complementation, particularly in relatively larger joints such as wrists. Another possible explanation for this is that grade 1 GS synovial hyperplasia should be considered as non-pathologic findings. Witt et al. illustrated that grade 1 GS synovial hyperplasia can be detected in up to 15% of the joints in healthy people, indicative of its unspecific nature to RA [42]. Generally, the range of capsule distension in healthy individuals shows broad variations that may overlap with the pathologic findings on GS. With the borderline finding of GS, it is difficult to distinguish between a pathologic and a physiologic state. On the contrary, grade 2 and grade 3 GS findings are more likely clinically significant. Different from the wrists, GS ≥ 1 synovial hyperplasia showed the best consistency to tenderness and swelling for MCP and PIP joints, indicating grade 1 synovial hyperplasia may be pathological for these small and superficial joints.

One interesting finding in the present study was that PD ≥ 1 synovitis showed the best consistency with clinical tenderness and swelling for all 22 joints, implying any grade of PD should be considered clinical significant. Compared with GS, findings on PD are usually more clearly defined. Previous studies reported a high correlation between PD positivity and inflammatory cell infiltration or vascularity in synovial tissues (*r* = 0.84, *p* < 0.01) [43]. PD positivity is obviously related to histopathological activity in patients with RA. The existence of PD signal predicted clinical relapse and further radiographic progression at both the patient level and joint level [15].

Only a small portion of patients received NSAIDs and corticosteroids. NSAIDs and corticosteroids can decrease the inflammatory parameters such as tender and swollen joint counts. Furthermore, Zayat et al. have confirmed that the usage of NSAIDs may mask the GS and PD signal and result in lower scoring despite continuing disease activity [44]. Previous studies also suggested that clinical parameters, including STC and TJC, GS and PD synovitis [45, 46], and the tenosynovitis [47], can be improved dramatically after the treatment of corticosteroids. Taken these together, these agents could improve both the clinical signs and inflammation detected by ultrasound, which could not substantially influence the results of this study.

There are some limitations of the study. Firstly, due to the absence of follow-up data, it is unclear whether grade 1 GS is associated with radiological progression. Clinical significance of grade 1 GS is required to address in further prospective studies. Secondly, we did not analyze the association of joint tenderness and swelling with other situations which may contribute these signs, such as edema, effusion, or osteophytes. Thirdly, either clinical signs or ultrasound examination is not a direct way to accurately assess the joint inflammation. But till now, imaging techniques especially ultrasound is a sensitive and convenient tool which is widely used in patients with RA.

## Conclusions

Joint swelling showed better agreement with US-determined inflammation than tenderness for MCP and PIP joints, while the opposite for wrists. Ultrasound synovitis had better agreement with clinical signs than tenosynovitis/peritendinitis. Both tenderness and swelling are more likely to correspond to GS ≥ 2 for wrists, GS ≥ 1 for MCP and PIP joints, and PD ≥ 1 for any joint.

## Abbreviations

ACPA: Anti-citrullinated peptide antibody
ACR: American College of Rheumatology
bDMARD: Biological disease-modified anti-rheumatic drugs
CDAI: Clinical Disease Activity Index
CRP: C-reactive protein
csDMARDs: Conventional synthetic disease-modified anti-rheumatic drugs
DAS28-CRP: Disease Activity Score based on 28 joint count and CRP
DAS28-ESR: Disease Activity Score based on 28 joint count and ESR
EGA: Evaluator’s global assessment
ESR: Erythrocyte sedimentation rate
EULAR: European League Against Rheumatism
GS: Gray scale
HAQ DI: Health Assessment Questionnaire disability index
ICC: Intra-class correlation coefficient
IQR: Interquartile range
MCP: Metacarpophalangeal
NSAIDs: Non-steroidal anti-inflammatory drugs
OMERACT: Outcome Measures in Rheumatology Clinical Trials
PD: Power Doppler
PE: Physical examination
PE−/US+: Non-tender/swollen joints but with US-detected inflammation
PE+/US−: Tender/swollen joints without US-determined inflammation
PGA: Patient’s global assessment
PIP: Proximal interphalangeal
PRF: Pulse repetition frequency
RA: Rheumatoid arthritis
RF: Rheumatoid factor
SD: Standard deviation
SDAI: Simplified Disease Activity Index
SJC-22: Swollen joint count in bilateral wrists, MCPs and PIPs joints
TJC-22: Tender joint count in bilateral wrists, MCPs and PIPs joints
US: Ultrasound
*κ*: Kappa

## References

[1] Grassi W, De Angelis R, Lamanna G, Cervini C (1998). The clinical features of rheumatoid arthritis. Eur J Radiol.

[2] van der Heijde DM, van ‘t Hof M, van Riel PL, van de Putte LB (1993). Development of a disease activity score based on judgment in clinical practice by rheumatologists. J Rheumatol.

[3] Smolen JS, Aletaha D (2014). Scores for all seasons: SDAI and CDAI. Clin Exp Rheumatol.

[4] van Gestel A, van Riel P (1996). American College of Rheumatology preliminary definition of improvement in rheumatoid arthritis: comment on the article by Felson et al. Arthritis Rheum.

[5] Felson DT, Smolen JS, Wells G, Zhang B, van Tuyl LH, Funovits J (2011). American College of Rheumatology/European League Against Rheumatism provisional definition of remission in rheumatoid arthritis for clinical trials. Arthritis Rheum.

[6] Kane D, Balint PV, Sturrock RD (2003). Ultrasonography is superior to clinical examination in the detection and localization of knee joint effusion in rheumatoid arthritis. J Rheumatol.

[7] Naredo E, Bonilla G, Gamero F, Uson J, Carmona L, Laffon A (2005). Assessment of inflammatory activity in rheumatoid arthritis: a comparative study of clinical evaluation with grey scale and power Doppler ultrasonography. Ann Rheum Dis.

[8] Brown AK, Conaghan PG, Karim Z, Quinn MA, Ikeda K, Peterfy CG (2008). An explanation for the apparent dissociation between clinical remission and continued structural deterioration in rheumatoid arthritis. Arthritis Rheum.

[9] Backhaus M, Kamradt T, Sandrock D, Loreck D, Fritz J, Wolf KJ (1999). Arthritis of the finger joints: a comprehensive approach comparing conventional radiography, scintigraphy, ultrasound, and contrast-enhanced magnetic resonance imaging. Arthritis Rheum.

[10] Naredo E, Collado P, Cruz A, Palop MJ, Cabero F, Richi P (2007). Longitudinal power Doppler ultrasonographic assessment of joint inflammatory activity in early rheumatoid arthritis: predictive value in disease activity and radiologic progression. Arthritis Rheum.

[11] Szkudlarek M, Court-Payen M, Jacobsen S, Klarlund M, Thomsen HS, Ostergaard M (2003). Interobserver agreement in ultrasonography of the finger and toe joints in rheumatoid arthritis. Arthritis Rheum.

[12] Foltz V, Gandjbakhch F, Etchepare F, Rosenberg C, Tanguy ML, Rozenberg S (2012). Power Doppler ultrasound, but not low-field magnetic resonance imaging, predicts relapse and radiographic disease progression in rheumatoid arthritis patients with low levels of disease activity. Arthritis Rheum.

[13] Ogishima H, Tsuboi H, Umeda N, Horikoshi M, Kondo Y, Sugihara M (2014). Analysis of subclinical synovitis detected by ultrasonography and low-field magnetic resonance imaging in patients with rheumatoid arthritis. Mod Rheumatol.

[14] Nguyen H, Ruyssen-Witrand A, Gandjbakhch F, Constantin A, Foltz V, Cantagrel A (2014). Prevalence of ultrasound-detected residual synovitis and risk of relapse and structural progression in rheumatoid arthritis patients in clinical remission: a systematic review and meta-analysis. Rheumatology (Oxford).

[15] Han J, Geng Y, Deng X, Zhang Z (2016). Subclinical synovitis assessed by ultrasound predicts flare and progressive bone erosion in rheumatoid arthritis patients with clinical remission: a systematic review and metaanalysis. J Rheumatol.

[16] Schmidt WA, Schmidt H, Schicke B, Gromnica-Ihle E (2004). Standard reference values for musculoskeletal ultrasonography. Ann Rheum Dis.

[17] Ellegaard K, Torp-Pedersen S, Holm CC, Danneskiold-Samsoe B, Bliddal H (2007). Ultrasound in finger joints: findings in normal subjects and pitfalls in the diagnosis of synovial disease. Ultraschall Med.

[18] Szkudlarek M, Wakefield RJ, Backhaus M, Terslev L (2012). The discriminatory capacity of ultrasound in rheumatoid arthritis: active vs inactive, early vs advanced, and more. Rheumatology (Oxford).

[19] Millot F, Clavel G, Etchepare F, Gandjbakhch F, Grados F, Saraux A (2011). Musculoskeletal ultrasonography in healthy subjects and ultrasound criteria for early arthritis (the ESPOIR cohort). J Rheumatol.

[20] Terslev L, Torp-Pedersen S, Qvistgaard E, von der Recke P, Bliddal H (2004). Doppler ultrasound findings in healthy wrists and finger joints. Ann Rheum Dis.

[21] Cyteval C (2009). Doppler ultrasonography and dynamic magnetic resonance imaging for assessment of synovitis in the hand and wrist of patients with rheumatoid arthritis. Semin Musculoskelet Radiol.

[22] Aletaha D, Neogi T, Silman AJ, Funovits J, Felson DT, Bingham CO (2010). 2010 Rheumatoid arthritis classification criteria: an American College of Rheumatology/European League Against Rheumatism collaborative initiative. Arthritis Rheum.

[23] Backhaus M, Burmester GR, Gerber T, Grassi W, Machold KP, Swen WA (2001). Guidelines for musculoskeletal ultrasound in rheumatology. Ann Rheum Dis.

[24] Wakefield RJ, Balint PV, Szkudlarek M, Filippucci E, Backhaus M, D'Agostino MA (2005). Musculoskeletal ultrasound including definitions for ultrasonographic pathology. J Rheumatol.

[25] Courvoisier DS, Agoritsas T, Glauser J, Michaud K, Wolfe F, Cantoni E (2012). Pain as an important predictor of psychosocial health in patients with rheumatoid arthritis. Arthritis Care Res (Hoboken)..

[26] Bjork M, Trupin L, Thyberg I, Katz P, Yelin E (2011). Differences in activity limitation, pain intensity, and global health in patients with rheumatoid arthritis in Sweden and the USA: a 5-year follow-up. Scand J Rheumatol.

[27] Ulus Y, Akyol Y, Tander B, Durmus D, Bilgici A, Kuru O (2011). Sleep quality in fibromyalgia and rheumatoid arthritis: associations with pain, fatigue, depression, and disease activity. Clin Exp Rheumatol.

[28] Basu N, Kaplan CM, Ichesco E, Larkin T, Harris RE, Murray A (2018). Neurobiologic features of fibromyalgia are also present among rheumatoid arthritis patients. Arthritis Rheumatol.

[29] McWilliams DF, Walsh DA (2016). Factors predicting pain and early discontinuation of tumour necrosis factor-alpha-inhibitors in people with rheumatoid arthritis: results from the British society for rheumatology biologics register. BMC Musculoskelet Disord.

[30] Wolfe F, Michaud K (2007). Assessment of pain in rheumatoid arthritis: minimal clinically significant difference, predictors, and the effect of anti-tumor necrosis factor therapy. J Rheumatol.

[31] Hart LE, Tugwell P, Buchanan WW, Norman GR, Grace EM, Southwell D (1985). Grading of tenderness as a source of interrater error in the Ritchie articular index. J Rheumatol.

[32] Lewis PA, O'Sullivan MM, Rumfeld WR, Coles EC, Jessop JD (1988). Significant changes in Ritchie scores. Br J Rheumatol.

[33] Thompson PW, Hart LE, Goldsmith CH, Spector TD, Bell MJ, Ramsden MF (1991). Comparison of four articular indices for use in clinical trials in rheumatoid arthritis: patient, order and observer variation. J Rheumatol.

[34] Prevoo ML, van ‘t Hof MA, Kuper HH, van Leeuwen MA, van de Putte LB, van Riel PL (1995). Modified disease activity scores that include twenty-eight-joint counts. Development and validation in a prospective longitudinal study of patients with rheumatoid arthritis. Arthritis Rheum.

[35] Ceponis A, Onishi M, Bluestein HG, Kalunian K, Townsend J, Kavanaugh A (2014). Utility of the ultrasound examination of the hand and wrist joints in the management of established rheumatoid arthritis. Arthritis Care Res (Hoboken).

[36] Krabben A, Stomp W, Huizinga TW, van der Heijde D, Bloem JL, Reijnierse M (2015). Concordance between inflammation at physical examination and on MRI in patients with early arthritis. Ann Rheum Dis.

[37] Felson D (2012). Defining remission in rheumatoid arthritis. Ann Rheum Dis.

[38] Lukas C, van der Heijde D, Fatenajad S, Landewe R (2010). Repair of erosions occurs almost exclusively in damaged joints without swelling. Ann Rheum Dis.

[39] Filer A, de Pablo P, Allen G, Nightingale P, Jordan A, Jobanputra P (2011). Utility of ultrasound joint counts in the prediction of rheumatoid arthritis in patients with very early synovitis. Ann Rheum Dis.

[40] Hussain Manik Z, George J, Sockalingam S (2016). Ultrasound assessment of synovial thickness of some of the metacarpophalangeal joints of hand in rheumatoid arthritis patients and the normal population. Scientifica (Cairo).

[41] Ohrndorf S, Boer AC, Boeters DM, Ten Brinck RM, Burmester GR, Kortekaas MC (2019). Do musculoskeletal ultrasound and magnetic resonance imaging identify synovitis and tenosynovitis at the same joints and tendons? A comparative study in early inflammatory arthritis and clinically suspect arthralgia. Arthritis Res Ther.

[42] Witt M, Mueller F, Nigg A, Reindl C, Leipe J, Proft F (2013). Relevance of grade 1 gray-scale ultrasound findings in wrists and small joints to the assessment of subclinical synovitis in rheumatoid arthritis. Arthritis Rheum.

[43] Takase K, Ohno S, Takeno M, Hama M, Kirino Y, Ihata A (2012). Simultaneous evaluation of long-lasting knee synovitis in patients undergoing arthroplasty by power Doppler ultrasonography and contrast-enhanced MRI in comparison with histopathology. Clin Exp Rheumatol.

[44] Zayat AS, Conaghan PG, Sharif M, Freeston JE, Wenham C, Hensor EM (2011). Do non-steroidal anti-inflammatory drugs have a significant effect on detection and grading of ultrasound-detected synovitis in patients with rheumatoid arthritis? Results from a randomised study. Ann Rheum Dis.

[45] Montecucco C, Todoerti M, Sakellariou G, Scire CA, Caporali R (2012). Low-dose oral prednisone improves clinical and ultrasonographic remission rates in early rheumatoid arthritis: results of a 12-month open-label randomised study. Arthritis Res Ther.

[46] Filippucci E, Farina A, Carotti M, Salaffi F, Grassi W (2004). Grey scale and power Doppler sonographic changes induced by intra-articular steroid injection treatment. Ann Rheum Dis.

[47] Ammitzboll-Danielsen M, Ostergaard M, Fana V, Glinatsi D, Dohn UM, Ornbjerg LM (2017). Intramuscular versus ultrasound-guided intratenosynovial glucocorticoid injection for tenosynovitis in patients with rheumatoid arthritis: a randomised, double-blind, controlled study. Ann Rheum Dis.

